# Editorial: Unveiling the next generation of cancer immunity & immunotherapy

**DOI:** 10.3389/fimmu.2025.1769324

**Published:** 2026-01-06

**Authors:** Mohanraj Sadasivam, Nethaji Muniraj, Chellappagounder Thangavel

**Affiliations:** 1 Division of Immunology, Department of Internal Medicine, University of Iowa Carver College of Medicine, Iowa, IA, United States; 2 Iowa City Veterans Affairs (VA) Health Care System, Iowa, IA, United States; 3 Children’s National Hospital, Center for Cancer and Immunology Research, Washington, DC, United States; 4 Center for Translational Medicine, Department of Medicine, Thomas Jefferson University, Philadelphia, PA, United States

**Keywords:** bites, cancer immunity and immunotherapy, cancer vaccine, CAR T cell therapy, checkpoint blockade, combination immunotherapy, cytokine therapy, immune checkpoint inhibitors

## Introduction

Over the past two decades, cancer immunology has shifted from a theoretical concept to a central pillar of modern oncology. Immune checkpoint inhibitors, adoptive cell therapies, oncolytic viruses, and personalized vaccines have revolutionized treatment for many malignancies. Yet, despite these remarkable advances, a substantial number of patients still fail to achieve durable responses, highlighting the persistent challenges of tumor heterogeneity, immune evasion, and the complex immunosuppressive tumor microenvironment (TME).

The Research Topic: *Unveiling the Next Generation of Cancer Immunity & Immunotherapy* brings together a collection of articles that address these challenges head-on, offering insights into innovative strategies designed to enhance the effectiveness, safety, and precision of immunotherapies. Collectively, these works showcase the field’s dynamic evolution and hint at what the future may hold for cancer treatment.

## Next-generation therapeutic design

**Figure 1:**
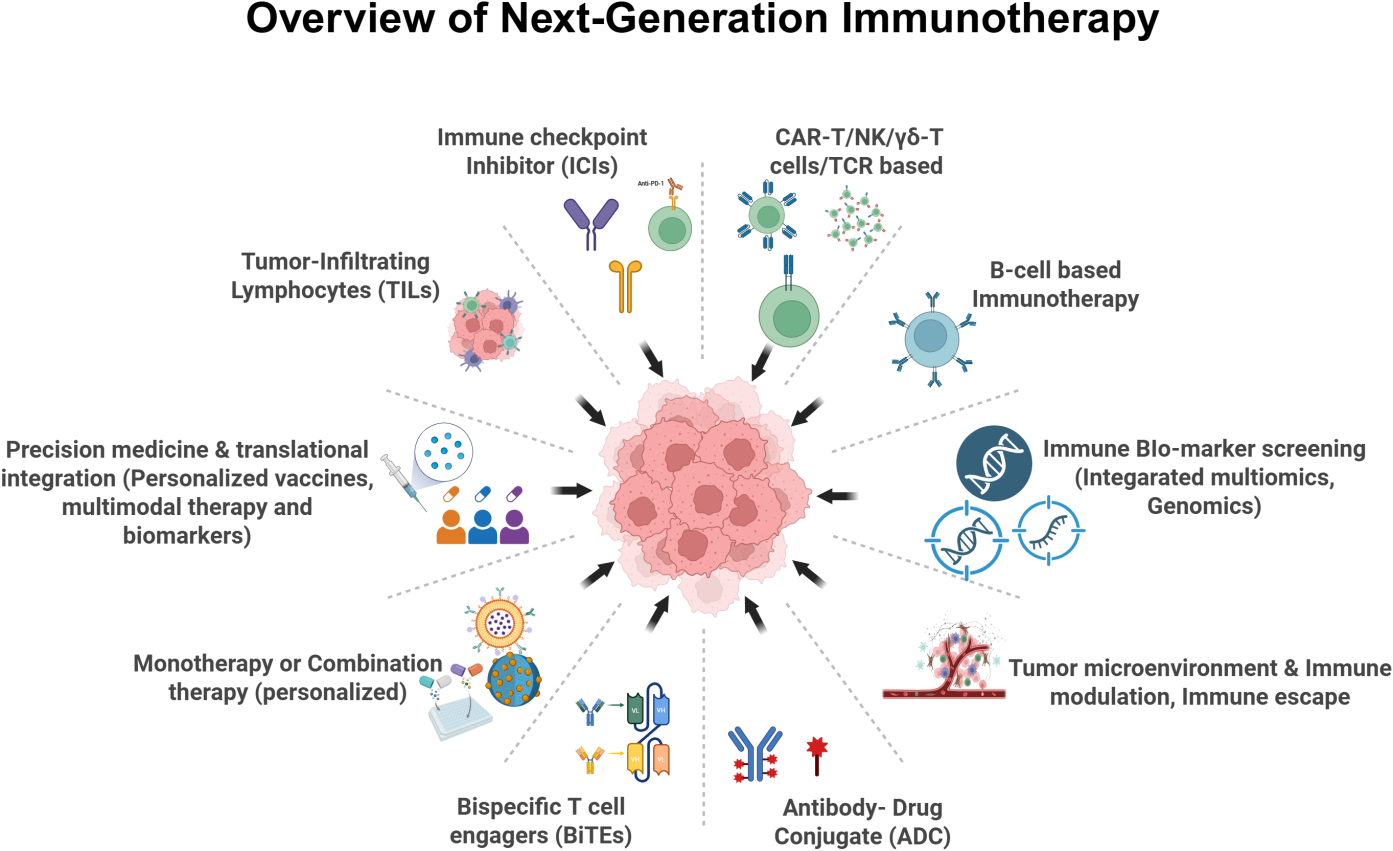
Overview of next-generation cancer immunotherapy: The schematic highlights next-generation immunotherapeutic approaches in cancer. Central to the figure is the tumor mass, surrounded by diverse immune-based strategies targeting tumor cells, including immune checkpoint inhibitors, adoptive cell therapies (CAR T, CAR NK, γδ T, and TCR-engineered cells), tumor-infiltrating lymphocytes, bispecific T cell engagers, antibody–drug conjugates, and B cell–based immunotherapies. The figure also illustrates tumor microenvironment modulation, immune escape mechanisms, biomarker-driven patient stratification, and precision medicine approaches that support personalized and combination immunotherapy.

A central theme across the Research Topic is the rise of multi-target and next-generation immunotherapies (**Figure 1**). Traditional monotherapies, while effective for some, often fall short due to tumor escape mechanisms. Dual-target strategies, for instance, seek to overcome this limitation by simultaneously modulating multiple pathways.

The review ‘*Unlocking new horizons in oncology: Ivonescimab’s dual-target approach to anti-VEGF/PD-1(L1) therapy’* exemplifies this approach. By targeting both angiogenesis and immune checkpoints, ivonescimab addresses the structural and immunological barriers that tumors employ to resist therapy. This dual-target strategy illustrates the power of rationally engineered biologics in maximizing immune responses while reducing the likelihood of resistance.

Similarly, ‘*Dual targeting of BCMA and SLAMF7 with the CARtein system: chimeric antigen receptors with intein-mediated* sp*licing elicit* sp*ecific T cell activation against multiple myeloma’* describes an advanced CAR T-cell platform in which intein-mediated protein splicing enables simultaneous recognition of two tumor antigens, resulting in enhanced and specific T-cell activation against multiple myeloma. This strategy aligns with the broader development of dual-target CAR approaches, such as CD19/CD22 and HER2/IL13Rα2 CARs, which are designed to improve antitumor efficacy and reduce antigen escape. Together, these innovations enhance target specificity, mitigate antigen escape, and exemplify the increasing sophistication of engineered cellular therapies, highlighting how advanced design principles can enable highly personalized, tumor-specific immunotherapeutic interventions.

The development of multi-functional antibodies further expands the therapeutic toolkit. ‘*Discovery and preclinical evaluation of BPB-101: a novel triple-functional bispecific antibody targeting GARP-TGF-β complex/SLC, free TGF-β and PD-L1’* highlights how one molecule can simultaneously disrupt several immunosuppressive pathways. By integrating checkpoint inhibition with modulation of TGF-β signaling, these approaches address multiple layers of tumor immune evasion in a single intervention.

## Reimagining the tumor microenvironment

Equally important are studies focusing on the tumor microenvironment, which plays a decisive role in shaping immunotherapy outcomes. The study ‘*Effect of bispecific recombinant oncolytic adenovirus carrying apoptin on apoptosis of MCF-7 cells’* demonstrates how engineered viruses can selectively eliminate tumor cells while simultaneously stimulating immune recognition, effectively converting “cold” tumors into “hot” ones more amenable to immune attack.

The review ‘*Revisiting the role of cancer-associated fibroblasts in tumor microenvironment’* reminds us that stromal cells are not passive bystanders. Cancer-associated fibroblasts actively shape immune cell infiltration, secrete regulatory cytokines, and influence extracellular matrix composition. Therapeutic strategies targeting or reprogramming these fibroblasts may enhance responses to both cellular and humoral immunotherapies.

Lymphoid structures also play a critical role, as highlighted in ‘*Immune microenvironment of tumor-draining lymph nodes: insights for immunotherapy’*. Tumor-draining lymph nodes orchestrate systemic immune responses, and their manipulation can significantly influence therapy outcomes. Understanding these hubs may allow us to improve T-cell priming, enhance trafficking, and ultimately improve clinical efficacy.

## Translational and clinical perspectives

Several contributions underscore the translation of mechanistic discoveries into clinical application. ‘*Personalized peptide-based immunization in an advanced-stage prostate cancer patient with bone metastasis’* illustrates how individualized immunotherapy can be practically implemented, even in late-stage disease. By designing vaccines tailored to a patient’s unique antigenic landscape, this work emphasizes the promise of precision medicine.

Additionally, ‘*Local radiotherapy in extensive-stage small-cell lung cancer sustainably boosts the clinical benefit of first-line immunotherapy’* highlights the power of combination strategies. Radiotherapy, traditionally seen as cytotoxic, can act synergistically with immunotherapy, promoting antigen release and immune activation. These studies collectively argue that next-generation immunotherapy is most effective when combined with other modalities.

## Emerging frontiers

Immunotherapy is undergoing a significant evolution, extending beyond its established role in oncology. CAR T-cell therapy, which has achieved notable clinical success in hematologic malignancies, is now being actively investigated for solid tumors as well as autoimmune and inflammatory diseases, reflecting growing confidence in the flexibility and translational potential of this platform. Although CD19 and BCMA remain the only approved CAR T-cell targets, an expanding repertoire of emerging antigens—including CD70, CD123, IL13Rα2, MUC1, NCAM, GD2, B7-H3, HER2, EGFRvIII, and mesothelin—is broadening the therapeutic landscape and enabling applications across solid and other non-hematologic tumors. The article ‘*Expanding the horizon of CAR T cell therapy: from cancer treatment to autoimmune diseases and beyond’* highlights how mechanistic and clinical insights gained in oncology can inform CAR T-cell strategies for autoimmune and inflammatory conditions, while ‘*B7-H3 in glioblastoma and beyond: significance and therapeutic strategies’* underscores the promise of alternative immune checkpoints as therapeutic targets for tumors resistant to conventional PD-1/PD-L1–based therapies. These insights point to a future where immunotherapy is not only more effective but also broader in scope and applicability.

## Synthesis and future directions

Across these studies, four themes emerge:

1. Innovative therapeutic design — the development of multi-target biologics, CAR-T/NK cells, and oncolytic viruses.
2. Tumor microenvironment modulation — understanding and manipulating stromal and lymphoid components to enhance immune response.
3. Precision immunotherapy — leveraging biomarkers, patient-specific vaccines, and adaptive strategies to maximize benefit.
4. Translational and clinical integration — combining conventional therapies, like radiotherapy, with advanced immunotherapies to achieve durable responses.

The field is moving from single-agent interventions toward multidimensional strategies that address tumor biology and immune contexture holistically. While challenges such as heterogeneity, resistance, toxicity, and access remain, the integration of mechanistic insights with translational innovation promises to deliver more durable, effective, and personalized therapies.

## Concluding remarks

The Research Topic ‘*Unveiling the Next Generation of Cancer Immunity & Immunotherapy’* provides a panoramic view of a rapidly evolving field. From cutting-edge therapeutic design and sophisticated cellular platforms to a nuanced understanding of the tumor microenvironment and clinical translation, these articles collectively chart the trajectory of next-generation immunotherapy.

By integrating molecular, cellular, and systemic perspectives, this Research Topic offers both a snapshot of current progress and a roadmap for future innovation. The challenge ahead is to translate these discoveries into durable, broadly applicable treatments that improve patient outcomes. These insights collectively indicate that, with ongoing cross-disciplinary integration, the field is well positioned to advance toward this outcome.

