# Correction: Editorial: Unveiling the next generation of cancer immunity & immunotherapy

**DOI:** 10.3389/fimmu.2026.1784505

**Published:** 2026-02-12

**Authors:** Mohanraj Sadasivam, Nethaji Muniraj, Chellappagounder Thangavel

**Affiliations:** 1 Division of Immunology, Department of Internal Medicine, University of Iowa Carver College of Medicine, Iowa, IA, United States; 2 Iowa City Veterans Affairs (VA) Health Care System, Iowa, IA, United States; 3 Children’s National Hospital, Center for Cancer and Immunology Research, Washington, DC, United States; 4 Center for Translational Medicine, Department of Medicine, Thomas Jefferson University, Philadelphia, PA, United States

**Keywords:** bites, cancer immunity and immunotherapy, cancer vaccine, CAR T cell therapy, checkpoint blockade, combination immunotherapy, cytokine therapy, immune checkpoint inhibitors

In the published article, the equal contribution statement was not applied to all authors. The correct statement appears below:

Mohanraj Sadasivam1†*, Nethaji Muniraj^2^†^*^ and Chellappagounder Thangavel^3^†^*^

“†These authors contributed equally to this work and share the first authorship”

The original version of this article has been updated.

